# Genome-Wide Analysis of *VILLIN* Gene Family Associated with Stress Responses in Cotton (*Gossypium* spp.)

**DOI:** 10.3390/cimb46030146

**Published:** 2024-03-11

**Authors:** Akash Deep, Dhananjay K. Pandey

**Affiliations:** Amity Institute of Biotechnology, Amity University Jharkhand, Ranchi 835303, India; akashaddeep5@gmail.com

**Keywords:** *VILLIN* gene (*VLN*), *Gossypium*, genome-wide characterization, cis-elements, gene expression, environmental stresses

## Abstract

The VILLIN (VLN) protein plays a crucial role in regulating the actin cytoskeleton, which is involved in numerous developmental processes, and is crucial for plant responses to both biotic and abiotic factors. Although various plants have been studied to understand the *VLN* gene family and its potential functions, there has been limited exploration of *VLN* genes in *Gossypium* and fiber crops. In the present study, we characterized 94 VLNs from *Gossypium* species and 101 VLNs from related higher plants such as *Oryza sativa* and *Zea mays* and some fungal, algal, and animal species. By combining these VLN sequences with other *Gossypium* spp., we classified the *VLN* gene family into three distinct groups, based on their phylogenetic relationships. A more in-depth examination of *Gossypium hirsutum VLNs* revealed that 14 *GhVLNs* were distributed across 12 of the 26 chromosomes. These genes exhibit specific structures and protein motifs corresponding to their respective groups. *GhVLN* promoters are enriched with cis-elements related to abiotic stress responses, hormonal signals, and developmental processes. Notably, a significant number of cis-elements were associated with the light responses. Additionally, our analysis of gene-expression patterns indicated that most *GhVLNs* were expressed in various tissues, with certain members exhibiting particularly high expression levels in sepals, stems, and tori, as well as in stress responses. The present study potentially provides fundamental insights into the *VLN* gene family and could serve as a valuable reference for further elucidating the diverse functions of *VLN* genes in cotton.

## 1. Introduction

In plant cells, the actin cytoskeleton is a complex and dynamic network that actively participates in several crucial activities, including signal transduction, vesicle trafficking, cell expansion, cell division, organelle movement, stomatal opening, and cytoplasmic streaming [1]. The diverse gelsolin/villin/fragmin superfamily, as well as nucleating proteins such as Formin and Arp2/3, monomer-binding protein profilin, severing/depolymerizing proteins ADF, and cofilin, are just a few actin-binding proteins (ABPs) that regulate cellular and cytoskeletal dynamics [2,3]. At certain time points and sites, ABPs control the assembly and disassembly of monomeric (G-actin) and filamentous actin (F-actin). Functional investigation of several ABPs has been performed in *Arabidopsis*. For instance, the loss of Actin-Depolymerizing Factor 5 (*AtADF5*) function has been shown to decrease drought tolerance and disrupt stomata closure due to abnormal actin dynamics [4]. The knockdown of *FORMIN3* (*AtFH3*) via RNA interference has been observed to reduce actin-cable abundance and affects pollen-tube growth [5]. Profilins (PRFs) are cytosolic proteins consisting of 129–133 amino acids with a molecular weight of 12–15 kDa and play a crucial role in regulating the cell cytoskeleton architecture, primarily through the modulation of actin polymerization [6,7,8]. These actin-binding proteins possess conserved profilin–actin interacting regions (PAINRs), which are essential for actin polymerization or depolymerization processes [7,8]. Recent studies have investigated their functional roles in root elongation, leaf morphology, epidermal expansion, flowering time, and seed germination across various plant species [9,10,11]. Fimbrin, another well-known ABP, also plays a role in the regulation of actin dynamics. In the atfim4/atfim5 double mutant, there were significant changes in the morphology and length of root hair [12]. Collectively, these studies indicated that ABPs are crucial for plant development and exert their effects by regulating actin dynamics.

VLN, a member of the villin/gelsolin/fragmin actin-binding protein (ABP) superfamily, is a key regulator of both actin stability and dynamics [5,13,14,15]. The regulatory functions of the VLN proteins are intricately linked to their structural characteristics. Six gelsolin home domains (G1-G6) at the N-terminus and a headpiece domain (VHP) at the C-terminus, each containing two conserved actin-binding domains, distinguish VLN proteins from other actin-binding domains (ABDs). ABD2 in the VHP area bundles actin filaments together, whereas ABD1, which spans G1 and G2, binds to two neighboring actin monomers inside the filament [16,17,18]. Additionally, VLN proteins possess calcium ion (Ca^2+^)-binding sites, which vary in type and number among different VLN variants. VLNs can bind to F-actin either independently or in a Ca^2+^/calmodulin-dependent manner. These features allow VLN proteins to alter the dynamics and organization of actin filaments, which helps create highly fibrillar actin structures [19,20,21,22]. In plant biology, the first functionally characterized VLN homologs were identified in *Lilium brownii* and were named 115-ABP and 135-ABP. These proteins bind to the F-actin [23,24,25]. Further investigations have revealed that 135-ABP plays a critical role in regulating the arrangement of F-actin in pollen tubes as well as the cytoplasmic architecture and actin-filament organization in root hairs [19,25]. There are five *VLN* genes in *Arabidopsis*, which is a model plant species. Although these genes are widely expressed in various plant tissues [26,27,28,29], only a subset of them has been functionally characterized. Specifically, the loss of *AtVLN1* and *AtVLN4* function has been associated with longer and shorter root hairs, respectively, indicating their distinct roles in the regulation of root-hair growth in *Arabidopsis* [30]. *AtVLN2* and *AtVLN3* have been implicated in normal plant development and organ morphogenesis [31,32], while *AtVLN5* is essential for pollen-tube growth [33]. Collectively, these studies underscore the vital role of VLNs in plant development through the regulation of various aspects of actin cytoskeleton and actin dynamics. To date, there has been limited research on VLN proteins in the *Gossypium* species. Several other actin-binding proteins (ABPs) and related proteins that may play a role in cytoskeletal dynamics and stress response activities have been investigated in *Gossypium* species, such as *profilin*, *LIM*, and *DUF668* [3,34,35].

In the present study, 14 *VLN* genes were identified in the *Gossypium hirsutum* genome. Subsequent analyses encompassed the examination of gene structures, conserved domains, phylogenetic relationships, chromosomal locations, cis-regulatory elements, gene synteny, and expression patterns. The present study provides comprehensive and valuable systematic insights that might be useful for the extended exploration of the functional aspects of *GhVLN* in cotton.

## 2. Materials and Methods

### 2.1. Sequence Retrieval and Identification of Villin Gene Family

The VLN sequence was retrieved from *Arabidopsis thaliana* using the NCBI Protein Database (https://www.ncbi.nlm.nih.gov/protein) (accessed on 9 September 2023) [36] with the accession number BAA96955.1, as the sequence structure was analyzed by Sato et al. in the year 2000 [37]. To identify all the possible VLN *Gossypium hirsutum*, we used a BLAST model consisting of six gelsolins and one VHP of *Arabidopsis thaliana* in *the Gossypium hirsutum* protein database from the Phytozome 13 Web BLAST Search (*Gossypium hirsutum v2.1*, https://phytozome-next.jgi.doe.gov/) (accessed on 13 September 2023) [38], with the target type set as Proteome, Program—BLASTP. The Expect threshold was configured as (−1), and a total of 100 set alignments were specified. We retrieved 14 protein sequences, identified by their protein IDs, as hits. To predict the conserved domains within GhVLN, we conducted a Batch Conserved Domain search using the NCBI platform (https://www.ncbi.nlm.nih.gov/Structure/cdd/wrpsb.cgi) (accessed on 16 September 2023) [39]. This process also helped to eliminate redundant sequences, and we used the default settings throughout. We used the SMART tool (http://smart.embl-heidelberg.de/) (accessed on 13 September 2023) [40] to confirm the existence of both gelsolin and VHP domains.

### 2.2. Analysis of Protein Physiochemical Properties, Amino Acid Alignment, and Phylogenetic Investigation

We utilized the ExPASy online platform (https://web.expasy.org/protparam/) (accessed on 13 September 2023) [41] to estimate a range of physicochemical characteristics of *Gossypium hirsutum* VLN, such as chromosome number, molecular weight (MW), isoelectric point (pI), protein length (measured in amino acid count), and Grand Average of hydropathy (GRAVY). BaCelLo (https://busca.biocomp.unibo.it/bacello/) (accessed on 13 September 2023) [42] tool was used to predict the subcellular locations of the proteins. Subsequently, a subcellular localization heatmap was generated using WoLF PSORT (https://wolfpsort.hgc.jp/) (accessed on 16 September 2023) [43]. Amino acid alignment was performed using DNAMAN Version 10 (Lynnon Biosoft, 2018). Phylogenetic tree was generated by aligning sequences and applying the neighbor-joining (NJ) method within MEGA11 software [44]. This analysis incorporated 1000 bootstrap replicates and employed the Jones–Taylor–Thornton (JTT) model. The tree was further modified using Interactive Tree Of Life (iTOL) Version 6.8.1 (https://itol.embl.de/) (accessed on 10 November 2023) [45].

### 2.3. Analysis of Gene Structure, Identification of Conserved Motifs, and Conducting Conserved Domain Analysis

We obtained the gene sequences of the *Gossypium hirsutum* VLN family protein from the Phytozome website (*Gossypium hirsutum* v2.1, https://phytozome-next.jgi.doe.gov/) (accessed on 13 September 2023) [38], and these sequences were extracted using TBtools (version 2.003). To illustrate gene structures, we utilized GFF3 files. Additionally, online tool GSDS 2.0 (http://gsds.cbi.pku.edu.cn/) (accessed on 16 September 2023) [46] was employed to visually represent the exon/intron structures of the *GhVLN* genes. NCBI Batch Conserved Domain search (https://www.ncbi.nlm.nih.gov/Structure/cdd/wrpsb.cgi) (accessed on 16 September 2023) [39] was used to predict conserved domains. For motif analysis, we used the MEME Suite (https://meme-suite.org/meme/tools/meme) (accessed on 24 September 2023) [47] to identify 20 motifs. All data were visualized using TBtools.

### 2.4. Collinearity and Chromosomal Location Analysis of GhVLN Genes

We obtained complete genome data for *Gossypium hirsutum* and extracted *Arabidopsis thaliana* genome sequences based on their transcript IDs using the FASTA Extract tool in TBtools. A collinearity relationship was established between these two species using the One-Step MCScanX feature of TBtools, with BlastP configured with eight CPU threads, an E-value of 1 × 10^−10^, and a minimum of five blast hits. Subsequently, we combined the CTL, GFF, and collinearity files using the Dual Synteny Plot within MCScanX in TBtools. All the chromosome scaffolds were manually removed from the CTL file before using the Dual Synteny Plot. Finally, we constructed and visualized the collinear relationship between *Gossypium hirsutum* and *Arabidopsis thaliana* by using TBtools. Additionally, we used MG2C_v2.1 Web Suite (http://mg2c.iask.in/mg2c_v2.1/) (accessed on 24 October 2023) [48] to visualize the chromosomal location of *GhVLN*. This analysis was based on the transcript IDs, chromosome numbers, and chromosome lengths.

### 2.5. GhVLNs Structure Prediction and Protein–Protein Interation

The 3D structures of GhVLN proteins were predicted using the online tool ExPASy SWISS-MODEL (https://swissmodel.expasy.org/) (accessed on 9 October 2023) [49]. When predicting protein structures in three dimensions, several key parameters are essential to assess the quality of the generated models. These parameters included global model quality evaluation (GMQE), coverage, and sequence identity.

GMQE is a value that ranges between 0 and 1, where a score closer to 1 indicates a better-quality model. This reflected the overall reliability of the model used in the prediction process. Coverage, on the other hand, measures the extent to which the target protein sequence aligns with and is covered by the template protein sequence. Finally, the sequence identity indicates how well the amino acid sequences align and match the target and template proteins. A higher sequence identity signifies a more reliable model, contributing to a greater accuracy in predicting the 3D structure of the protein.

In our quest to understand the function of GhVLNs, we built protein interaction networks by utilizing the online tool STRING (https://string-db.org/) (accessed on 9 October 2023) [50], where we configured the Required Score to a medium confidence level of 0.4 and applied a medium FDR Stringency of 5 percent.

### 2.6. Measurement of Evolutionary Selection Pressure and Cis-Element Analysis

Pairs of genes with similar genetic relationships were chosen based on a phylogenetic tree alignment of sequences using the neighbor-joining (NJ) method within the MEGA11 software [44] and Interactive Tree Of Life (iTOL) Version 6.8.1 (https://itol.embl.de/) (accessed on 10 November 2023) [45]. Subsequently, the TBtools software was used to compute the values for Ka (non-synonymous rate), Ks (synonymous substitution), and Ka/Ks (evolutionary constraint). The divergence time (T) was determined using the formula T = Ks/(2 × 9.1 × 10^−9^) × 10^−6^ million years [51]. In general, if Ka/Ks < 1.0, it indicates purifying or negative selection, Ka/Ks = 1.0 represents neutral selection, and Ka/Ks > 1.0 signifies positive selection [52]. We conducted GhVLN cis-element prediction using Plant CARE (https://bioinformatics.psb.ugent.be/webtools/plantcare/html/) (accessed on 19 November 2023) [53] by analyzing a 3.5 kb sequence upstream of the coding region. The results were visualized using the BioSequence Structure Illustrator in the graphics section of TBtools.

### 2.7. Expression Patterns of GhVLNs in Different Tissues and Stress Conditions

We obtained the expression levels of *Gossypium hirsutum* RNA-Seq data, measured in Fragments Per Kilobase of transcript per million mapped reads (FPKM), for each *GhVLN* gene under various stress conditions, including drought, salt, heat, and cold stress, at different time points (0, 1, 3, 6, and 12 h). These data were extracted from the CottonRNA Database (PRJNA490626) (http://ipf.sustech.edu.cn/pub/cottonrna/) (accessed on 25 November 2023), and TBtools was used to visualize and present the gene-expression patterns based on the FPKM values.

## 3. Results

### 3.1. Sequence Retrieval of VLN Gene and Characterization of GhVLNs

**Figure 1:**
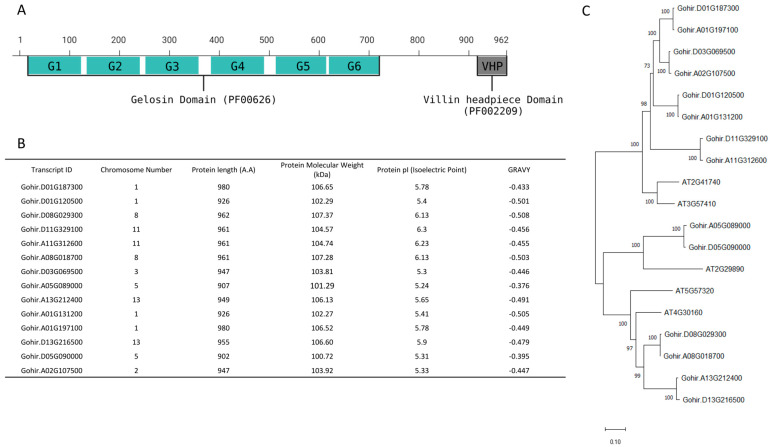
Isolation and identification of GhVLNs: (**A**) Gelsolin domain (PF00626) and the Villin-headpiece domain (PF02209)-conserved domains in GhVLNs. (**B**) Comprehensive assessment of physical and chemical attributes of GhVLNs. (**C**) Evolutionary linkage among between VLNs found in *Gossypium hirsutum* and *Arabidopsis thaliana*.

To identify the Villin (VLN) proteins in cotton, we utilized protein sequences containing gelsolin and villin-headpiece (VHP) domains as query sequences from the PFAM database. These sequences comprised six gelsolin domains and a villin-headpiece domain (Figure 1A).

We initiated a BLAST search against the *Gossypium hirsutum* protein database available on the Phytozome 13 website, followed by an NCBI Batch Conserved Domain search to eliminate redundant sequences. A total of 14 VLN proteins were identified in *Gossypium hirsutum*.

**Figure 2:**
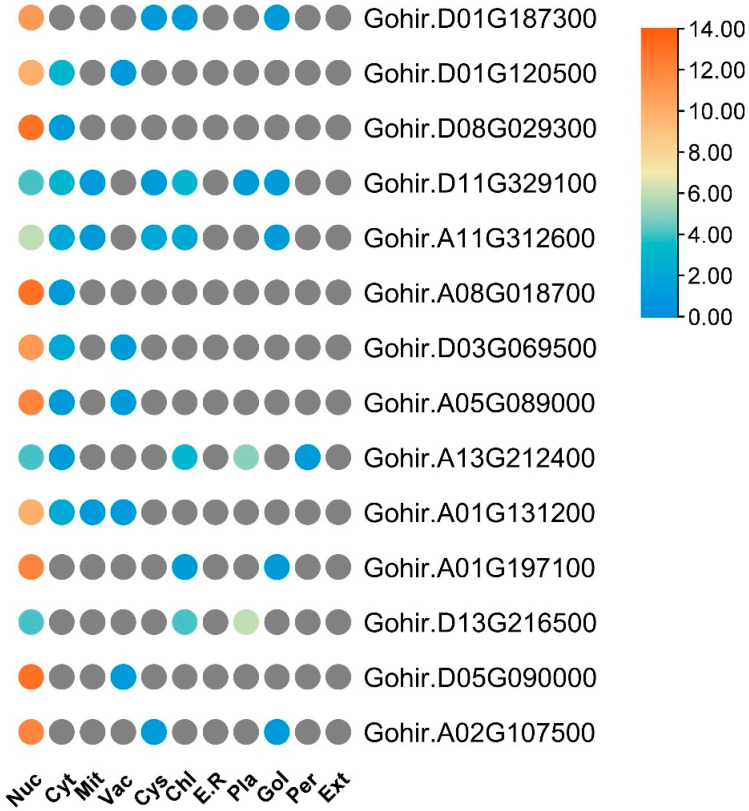
Prediction scores for WoLF PSORT plant data sets are presented, indicating subcellular localization probabilities for various compartments. Abbreviations such as ‘Nuc’ for Nuclear, ‘Cyt’ for cytosol, ‘Mit’ for mitochondria, ‘Vac’ for vacuole, ‘Cys’ for cytoskeleton, ‘Chl’ for chloroplast, ‘E.R’ for endoplasmic reticulum, ‘Pla’ for plasma membrane, ‘Gol’ for Golgi apparatus, ‘Per’ for peroxisome, and ‘Ext’ for extracellular, are utilized to denote specific cellular locations.

To obtain a more profound understanding of GhVLNs, we extensively examined their diverse physicochemical characteristics. These included chromosome number, amino acid protein length, isoelectric point (pI), molecular weight (MW), subcellular distribution, and grand average of hydropathy (GRAVY). GhVLN proteins are 902–980 amino acids in length. Their estimated molecular masses varied from 100.72 to 107.37 kDa, with Gohir.D01G187300 having the longest protein length (980 A.A) and Gohir.D08G029300 having the highest molecular mass (107.37 kDa) (Figure 1B). The predicted isoelectric points of the GhVLNs ranged from 5.24 to 6.23, with all pI values greater than 5.25. Additionally, initial predictions indicated that most GhVLNs were localized to the nucleus (Figure 2).

Interestingly, we observed that certain paralogous of GhVLNs sequences, such as Gohir.D01G187300 and Gohir.A01G197100, had identical protein lengths and isoelectric points but differed in molecular weights. Similarly, Gohir.D03G069500 and Gohir.A02G107500 shared the same protein length but exhibited slight variations in molecular weight and isoelectric point (Figure 1B).

**Figure 3:**
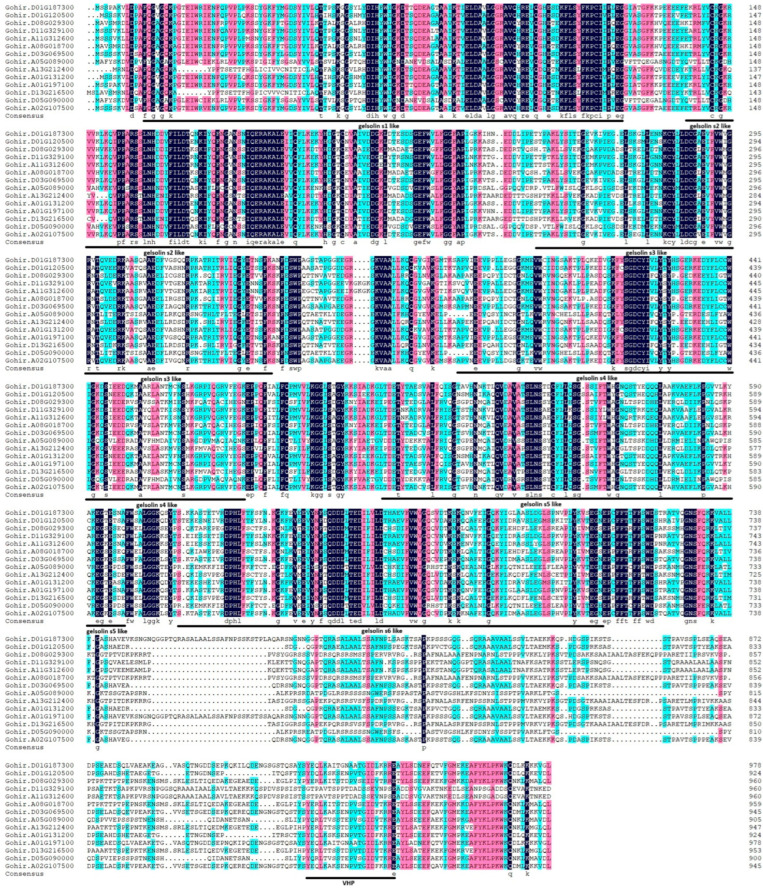
Amino acid alignment of GhVLN proteins reveals conserved domains, including G1–G6 and a VHP domain, depicted by distinct lines matching these domains across all sequences. This visual representation highlights the conservation of essential motifs within the GhVLN protein family. Different colors delineate the degrees of similarity among 14 VLN protein sequences: cyan shades denote similarity ranging from 50% to 74%, cherry red signifies similarity from 75% to 99%, and deep blue represents 100% similarity.

**Figure 4:**
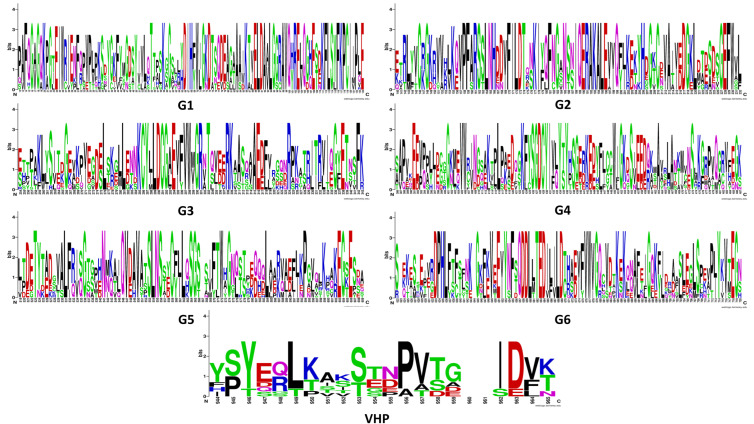
Conserved motifs identified within *GhVLN*s as G1–G6 and VHP are depicted. These conserved motifs serve as key structural and functional elements within the GhVLN protein family, potentially influencing various biological processes. The colors of amino acids correspond to their chemical properties; polar, basic, acidic, and hydrophobic amino acids are represented by green, blue, red, and black respectively.

Additionally, by employing the VLN protein sequence from *Arabidopsis thaliana* as a model, we conducted a multiple-sequence-alignment analysis of *Gossypium hirsutum*. This analysis revealed that all 14 GhVLNs featured six distinctive gelsolin domains and one headpiece domain (Figure 3 and Figure 4). These observations suggest that the existence of both shared and distinct protein functions and characteristics within GhVLNs may contribute to diversifying the *Villin* gene function in *Gossypium hirsutum*.

### 3.2. Phylogenetic Analysis of Gossypium Species and Their Orthologs from Other Model Species

To investigate the evolutionary connections between the GhVLN across various *Gossypium* species and their counterparts in some different species, we conducted a phylogenetic analysis. To compile candidate VLN protein sequences, we examined protein databases encompassing both lower and higher plants, including monocotyledons and dicotyledons and also some fungal, algal, and animal species. Candidate sequences were identified by querying the respective databases using the initial query sequences. We ensured that the selected proteins included the conserved gelsolin domain and used the headpiece domain for phylogenetic analysis.

In total, we obtained 94 VLN protein sequences distributed across various *Gossypium* species (Supplementary Table S1).

**Figure 5:**
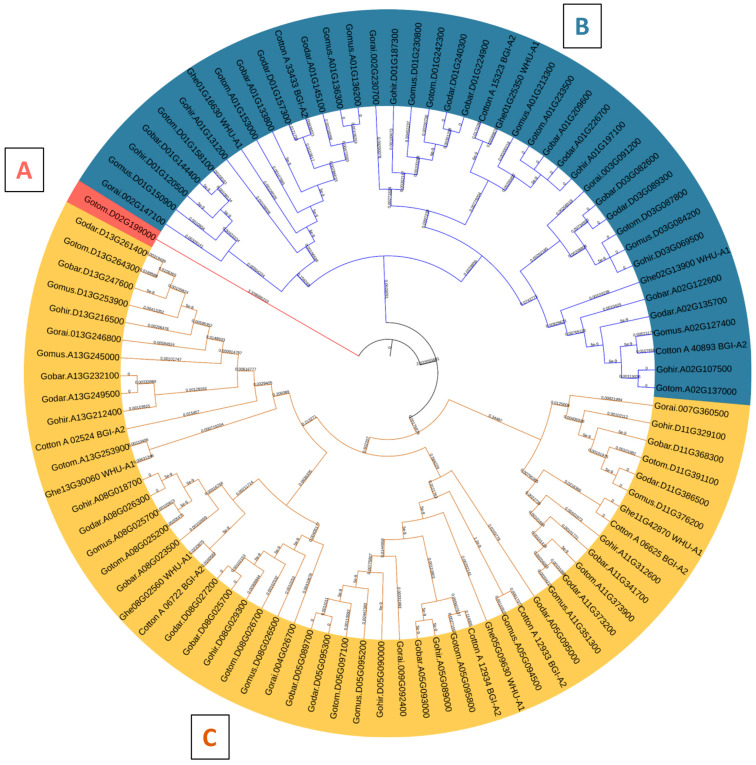
Phylogenetic analysis of *VLN* possessing sequences in different *Gossypium* species including 14 from *Gossypium hirsutum* (*Gohir*), 8 from *Gossypium arboretum* (*Cotton_A*), 14 from *Gossypium barbadense* (*Gobar*), 14 from *Gossypium darwinii* (*Godar*), 7 from *Gossypium herbaceum* (*Ghe*), 15 from *Gossypium mustelinum* (*Gomus*), 7 from *Gossypium rainmondii* (*Gorai*), and 15 from *Gossypium tomentosum* (*Gotom*) distributed into three distinct clades (**A**–**C**).

The phylogenetic analysis successfully classified the 94 VLN proteins into three discernible clades, designated as A, B, and C. Clade A was characterized by a solitary VLN protein, whereas Clades B and C exhibited larger memberships, comprising 40 and 53 VLN proteins, respectively (Figure 5). The phylogenetic tree revealed that Gotom.D02G199000, derived from *Gossypium tomentosum*, is the sole member of Clade A, exhibiting a notable divergence from the midroot point. Meanwhile, the remaining sequences displayed an uneven distribution across Clades B and C, hinting at intriguing evolutionary relationships (Figure 5).

**Figure 6:**
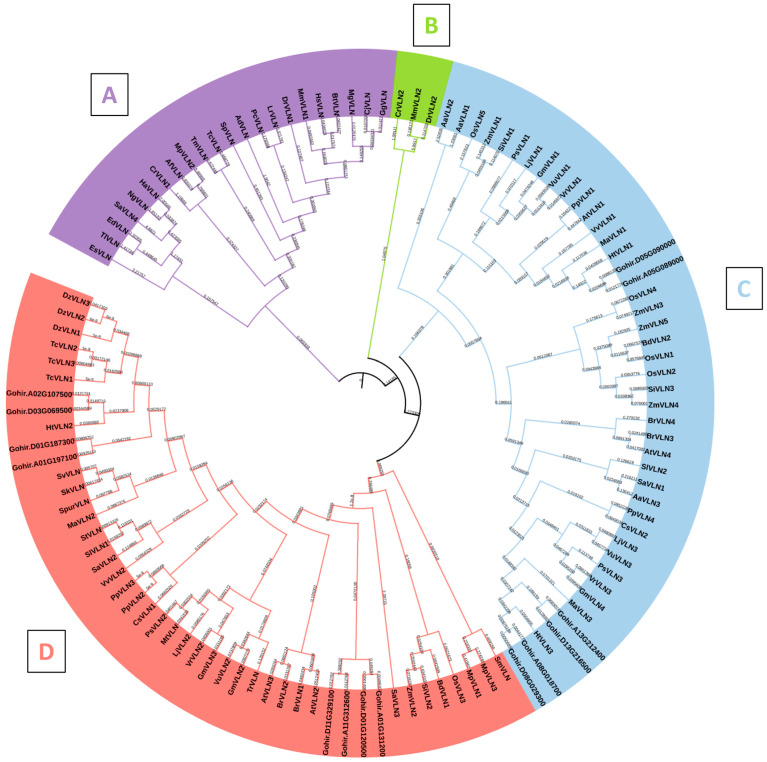
Phylogenetic analysis depicting the evolutionary relationships of the VLN protein from *Gossypium hirsutum* alongside diverse plant species, organized into four distinct clades (**A**–**D**), providing valuable insights into the evolutionary trajectory of this gene.

The phylogenetic analysis of GhVLNs, alongside VLN proteins from diverse representative species, resulted in the classification of 115 VLNs into four distinct clades: A, B, C, and D. Clade A comprised 22 VLN proteins, while Clades B, C, and D contained 3, 44, and 46 VLN protein sequences, respectively. These clades were systematically distributed based on their evolutionary resemblances (Figure 6). Villin protein sequences were sourced from various taxa, including Animalia (12 sequences), Fungi (3 sequences), Amoebozoa (3 sequences), Algae (4 sequences), and Plantae, further categorized into monocots (35 sequences) and dicots (58 sequences) (Supplementary Table S2). Interestingly, the number of VLN proteins varied among different species. Notably, a tree clade comprising Clades B, C, and D, which share a common ancestral origin, exhibited the maximum diversity in Villin gene evolution. This clade encompassed a range of species from lower to higher plants, as well as lower to higher classes of animals and fungi, underscoring the ancient origin of the *Villin* gene family. Clade B, the smallest clade with only three sequences, included the algal species *Chlamydomonas reinhardtii*, alongside animalia species such as *Mus musculus* and *Danio rerio*, suggesting a potential shared ancestry. Clade D emerged as the largest, comprising 46 sequences, predominantly from dicot plants, particularly within the Fabaceae family (16 species), and monocot plants, primarily within the Poaceae family (11 species). Notably, Clade D excluded species from the Animalia, Fungal, Algal, and Amoebozoa groups. Similarly, Clade C exhibited abundant representation from plant species, spanning monocot and dicot families (Figure 6). Within Clade A, there was a notable abundance of animalia species, including mammals (such as *Homo sapiens*, *Mus musculus*, and *Bos taurus*), Aves (such as *Gallus gallus*, *Coturnix japonica*, and *Meleagris gallopavo*), and aquatic species (such as *Labeo rohita*, *Danio rerio*, and *Strongylocentrotus purpuratus*). These species are found in similar subclades, suggesting a potential common ancestry with some Fungal and Amoebazoa species. Notably, only two plantae species (*Striga asiatica* and *Marchantia polymorpha*) are present in Clade A, and they share similar subclades with fungi, such as *Aspergillus fishcheri*, and certain algal species. Notably, it was found that sequences of *Gossypium hirstusum* were only found in Clades C and D (Figure 6), where all the higher plant species were present, and the *Gossypium hirstusum* sequences showed homology with its counterpart sequences only.

These results from the evolutionary analysis suggest that the *VLN* gene underwent divergence during the evolution of different species yet remained highly conserved in higher plants consisting of monocotyledons and dicotyledons.

### 3.3. Gene Structure Analysis of GhVLNs and the Conserved Domain and Motif Analysis of GhVLNs

**Figure 7:**
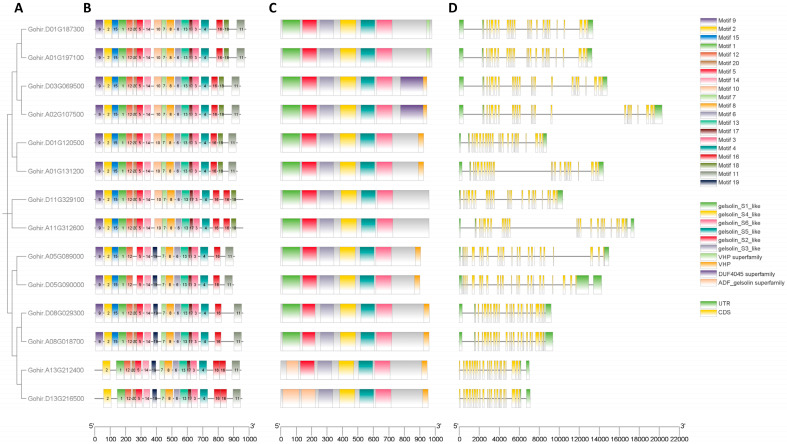
Gene structure of GhVLNs, motif, and conservative domain analysis of GhVLNs. (**A**) Intra-species phylogenetic tree of GhVLNs. (**B**) The motif composition of GhVLNs, with conserved motifs in *Gossypium* VLN proteins indicated by colored boxes. (**C**) Conserved domains of GhVLNs, with colored boxes representing the conserved protein domain. (**D**) The gene structure of *GhVLNs*; green boxes, yellow boxes, and black lines represent the UTR, exons, and introns, respectively.

To delve deeper into the structural characteristics of *GhVLNs* and the proteins they encode, we conducted a comprehensive examination of their gene structures, conserved motifs, and domains. Our analysis of *GhVLN* genes also involved investigating their exon/intron structures and identifying conserved motifs (Figure 7A–D). Notably, these genes exhibit a significant number of exons and introns, indicating extensive genomic sequences. The number of introns across the 14 *GhVLNs* varied from 21 to 24 (Figure 7D).

We also investigated the motifs and domains present in the GhVLN proteins to enhance our understanding of their conservation and diversification. Twenty distinct motifs were identified and labeled as Motifs 1–20. Additionally, our analysis showed conservation of the GhVLN domains, characterized by the presence of six gelsolin domains and a headpiece domain (VHP) (Figure 7C). Gene structural analyses collectively suggest that *GhVLNs* genes and their encoded proteins possess complementary structures, and these structures may explain their functional distinctions. This knowledge may provide a foundational structural framework for understanding the conversed gene functions.

The sequences corresponding to the 20 identified motifs vary in length and span from 21 to 50 amino acids (Table 1). Notably, the counts of these conserved motifs differed among GhVLN proteins, ranging from 16 to 19 motifs per protein (Figure 7B). Proteins identified by the transcript IDs Gohir.D08G029300, Gohir.A08G018700, Gohir.A05G089000, Gohir.A13G212400, Gohir.D13G216500, and Gohir.D05G090000 were found to be without motifs 10 and 18. However, these proteins exclusively contained motif 19. Additionally, Gohir.A13G212400 and Gohir.D13G216500 lacked motifs 9 and 15. Furthermore, Gohir.A05G089000 and Gohir.D05G090000 lacked motif 20, and Gohir.D11G329100 and Gohir.A11G312600 were devoid of motif 11. The DUF4045 superfamily domain was found in proteins Gohir.A02G107500 and Gohir.D03G069500 (Figure 7C). Most GhVLN proteins consist of six gelsolin homology domains (gelsolin s1 to s6-like) and a villin-headpiece domain (VHP), consistently, as mentioned elsewhere. However, it is important to note that exceptions exist, as Gohir.A13G212400 and Gohir.D13G216500 lacks the (G1) and (G1-2) homology domains, respectively, and two proteins, Gohir.D11G329100 and Gohir.A11G312600, do not possess the headpiece domain (VHP) (Figure 7C).

Hence, the gelsolin homology domains exhibited a remarkable degree of conservation within cotton VLNs. Furthermore, GhVLN orthologs that shared close evolutionary relationships displayed analogous motif architectures and *GhVLNs* exon/intron distribution patterns, highlighting similarities in their structural characteristics.

### 3.4. Collinearity and Chromosomal Location Analysis of GhVLNs Gene

**Figure 8:**
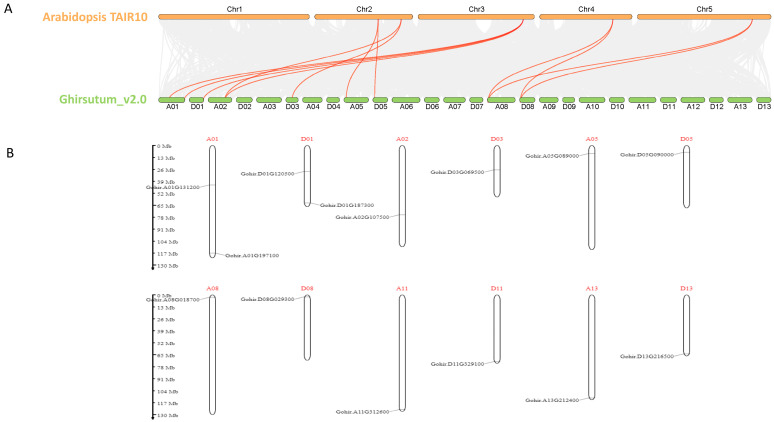
Analysis of collinearity and chromosomal positions of *GhVLNs*: (**A**) We chose the model plant *Arabidopsis thaliana* for establishing collinearity with *Gossypium hirsutum*. In the graphical representation, the gray lines in the backdrop indicate collinear blocks shared between *Arabidopsis thaliana* and *Gossypium hirsutum*, whereas the red lines denote collinear blocks associated with *VLN* genes. (**B**) This diagram displays the physical positions of *GhVLNs* on the chromosomes, with the scale on the left indicating the genomic length in megabases (Mb).

To delve deeper into the functional mechanisms of *Gossypium hirsutum VLNs*, we conducted a collinearity analysis using the genomes of *Arabidopsis thaliana* and *Gossypium hirsutum*. This analysis revealed 12 orthologous pairs of *VLNs* between the two genomes (Figure 8A). Importantly, it was observed that several *VLN* genes in *Gossypium hirsutum* displayed synteny with the same *VLN* gene in *Arabidopsis thaliana*, suggesting a scenario where *GhVLNs* may have originated from a common ancestor through duplication events during evolution. These results suggest that *VLNs* were relatively conserved between *Arabidopsis thaliana* and *Gossypium hirsutum* during the evolution of higher plants.

In the analysis of collinearity of *GhVLNs* with *Arabidopsis*, we observed that the *VLN* genes were unevenly distributed across 12 of the 26 chromosomes in *Gossypium hirsutum* (Figure 8A). *GhVLNs* are located at the chromosomal termini of A01, D01, A02, A05, D05, A08, D08, A11, D11, A13, and D13. However, on chromosomes A01, D02, and D03, VLN locations were near the centromeres of the chromosomes (Figure 8B).

### 3.5. Three-Dimensional Structure Prediction and Protein–Protein Interaction Network

In the analysis of 3D-structure prediction, three key parameters—Global Model Quality Evaluation (GMQE), coverage, and sequence identity—were employed to assess and select templates. A higher value for these parameters signifies greater accuracy, and the template with the highest score was chosen. In our study, GMQE values consistently fell within the range of 0.73 to 0.79, with a 100% coverage rate and sequence identity ranging from 70.12 to 99.38. These values indicate a high level of reliability for the selected templates.

**Figure 9:**
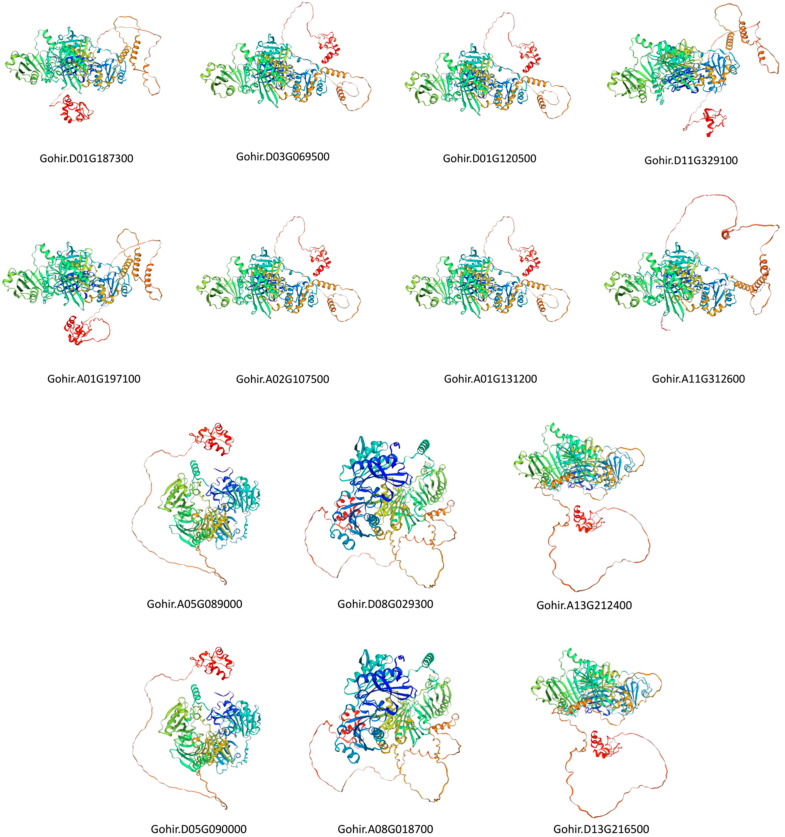
Three-dimensional prediction of GhVLN proteins by SWISS-MODEL. The rainbow color scheme provides a visual representation of different structural features within the protein, aiding in the interpretation of its predicted three-dimensional structure.

Furthermore, in the same subgroup, the prediction templates remained consistent, highlighting their evolutionary conservation, except in Groups 1 and 4. This underscores the reliability of the selected prediction templates. Overall, these results suggest that GhVLN proteins within the same subgroup exhibit highly similar spatial structures (Figure 9). Villin belongs to the gelsolin superfamily and functions as a protein involved in F-actin nucleation, crosslinking, severing, and capping. The villin headpiece is crucial for anchoring villin to F-actin, facilitating the process of crosslinking [16]. Detailed information regarding protein structure predictions, including metrics like Coverage, GMQE (Global Model Quality Estimation), and identity scores, are presented in (Supplementary Table S3).

To gain a deeper understanding of the role of the GhVLN protein family in plant development, we predicted protein-interaction networks and three-dimensional structures of all GhVLN proteins.

**Figure 10:**
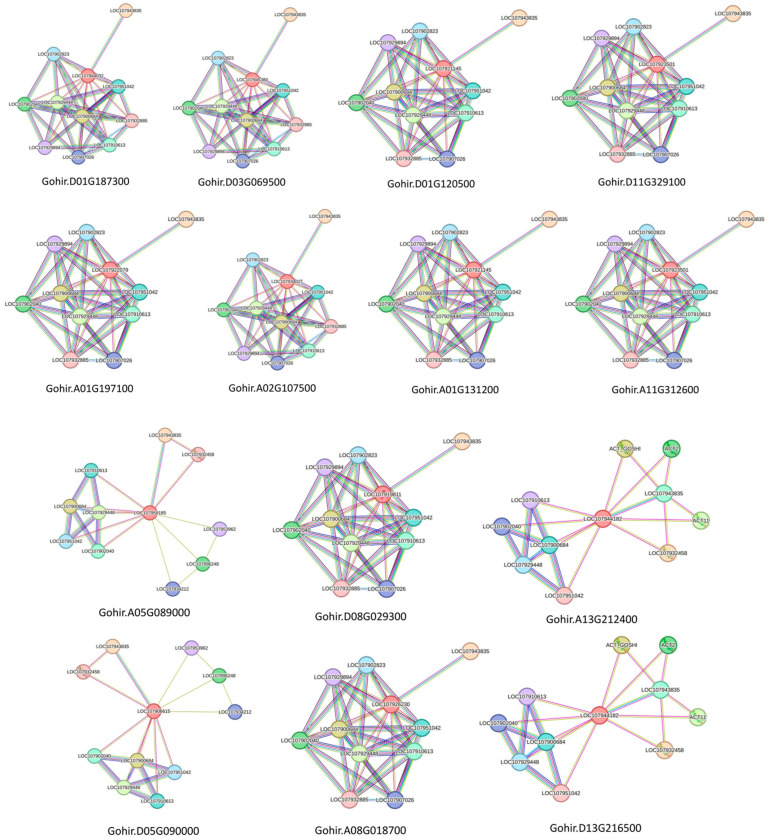
Protein–protein interactions among GhVLN proteins, where each node of different color represents an individual protein, and each edge signifies an interaction. The red loci represent the query GhVLN protein. Edges in the visualization are assigned colors corresponding to different types of evidence: known interactions from curated databases and experimentally determined interactions are depicted by cyan and pink lines, respectively. Predicted interactions derived from gene neighborhood, gene fusions, and gene co-occurrence are represented by green, red, and blue lines. Additionally, other evidence types obtained through text-mining, co-expression, and protein homology are indicated by dark yellow, black, and violet lines, respectively.

Our analysis revealed that GhVLN proteins primarily interact with various cytoskeletal proteins and Golgi function-related proteins and play roles in stress responses, including interactions with arabinogalactan proteins (AGPs), the conserved oligomeric Golgi complex (COG), and leucine-rich repeat extensin proteins (LRXs) (Figure 10). Additionally, GhVLN proteins engage with proteins related to development, stress responses, and hormones, suggesting their involvement in signaling response pathways. Detailed information regarding these protein interactions is listed in (Supplementary Table S4). Moreover, there were several other proteins, including LOC107907026, whose protein families have not yet been identified. Initial functional predictions suggest that these proteins also play crucial roles in various cellular physiological processes (Figure 10).

### 3.6. Analysis of Evolutionary Selection Pressure

Calculating Ka (non-synonymous substitution), Ks (synonymous substitution), and Ka/Ks (evolutionary selection pressure) is of significant importance in determining phylogenetic relationships and gaining insights into evolutionary dynamics within and among species [7]. The Ka/Ks ratio serves as a valuable indicator for assessing the selection pressure in recurrent events. When Ka/Ks is less than one, it signifies purifying selection; when it equals one, it indicates neutral selection; and when it exceeds one, it suggests a positive selection [7].

Therefore, we computed Ka/Ks values for collinear gene pairs within *Gossypium hirsutum* (Table 2). The Ka/Ks ratios for *GhVLN* gene pairs ranged from 0.096 to 0.305, with an average of 0.201. Notably, all Ka/Ks values for *GhVLN* gene pairs were less than one, implying that these genes evolved predominantly under the influence of purifying selection (Table 2).

The estimated divergence time for these seven gene pairs ranged from 1.565 million years ago (MYA) for Gohir.D01G187300-Gohir. A01G197100 to 3.160 (MYA) for Gohir.A13G212400-Gohir.D13G216500 with an average divergence time of approximately 2.396 (MYA) (Table 2).

### 3.7. Cis-Element and Expression Analysis of GhVLNs

**Figure 11:**
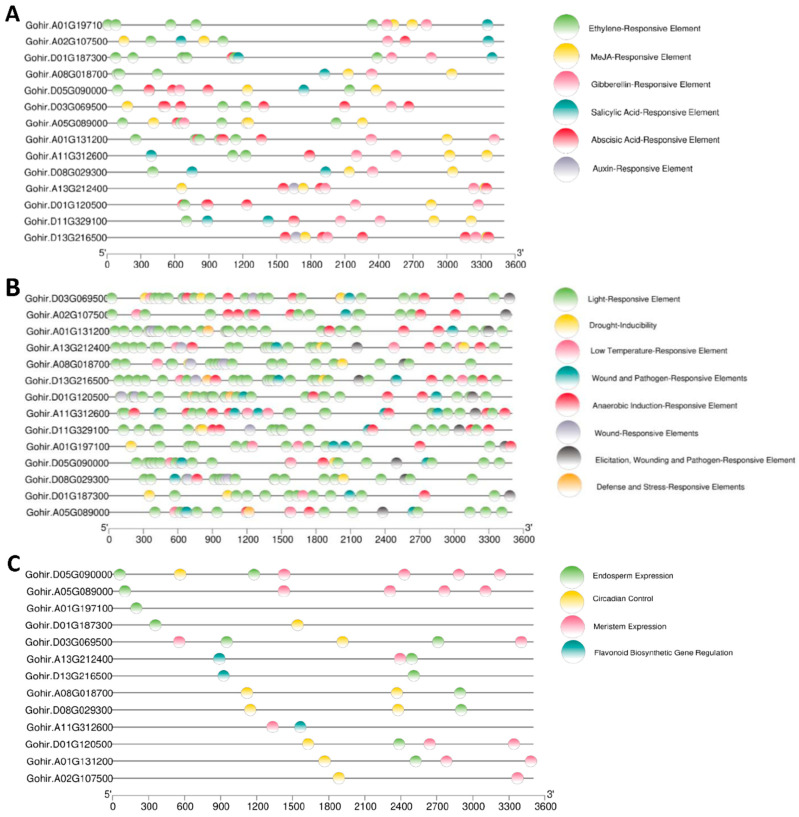
Cis-element analysis of (**A**) environmental stress responses, (**B**) hormonal biological processes, (**C**) meristem expression, circadian control, endosperm expression, and regulation of flavonoid biosynthetic genes.

To further explore the potential biological roles of *GhVLNs*, we conducted a comprehensive analysis of cis-elements present in the predicted promoter regions of these genes. We focused on a 3.5 kb segment located upstream of the *GhVLN* start codon, using it as the promoter region for our investigation. The identification of cis-elements in the *GhVLNs* promoter region was carried out using the online Plant CARE tool, and sequence illustrative plots were generated using TBtools (Figure 11A–C).

Our analysis revealed that the promoter region of *GhVLNs* contains a variety of cis-elements that are associated with key hormonal biological processes. These include elements linked to Abscisic Acid (ABRE), MeJA (TGACG-motif, TATC-box, and CGTCA-motif), Gibberellin (GARE-motif and P-box), Ethylene (ERE), Salicylic Acid (TCA-elements), and Auxin (TGA-elements) responsiveness (Figure 11A). Moreover, we observed the presence of cis-elements related to environmental stress responses, such as Anaerobic Induction (ARE), elicitation (box S), wound (WUN-motif), pathogen (W box), low temperature (LTR), drought inducibility (MBS), and light, defense, and stress (TC-rich repeats) responsiveness (Figure 11B).

It is worth noting that some motifs, such as Heat stress (HSE), Fungal Elicitor (Box-W1, W3), Dehydration, and Salt stress (DRE)-responsive elements, were absent in the *GhVLNs*’ promoter region. Notably, the most prominent elements were related to the light response. Furthermore, the promoter region contained cis-elements associated with meristem expression, circadian control, endosperm expression, and the regulation of flavonoid biosynthetic genes (Figure 11C).

This comprehensive cis-element analysis suggests that *GhVLNs* may be involved in a range of signaling pathways and biological processes. However, it is important to emphasize that, while these findings are intriguing, further research is needed to substantiate these speculations. Analyzing the gene function of *GhVLNs* in the context of their responses to external- and internal-environmental signals holds great promise for advancing our understanding in this area.

### 3.8. Gene-Expression Pattern Analysis in Different Organs of GhVLNs

**Figure 12:**
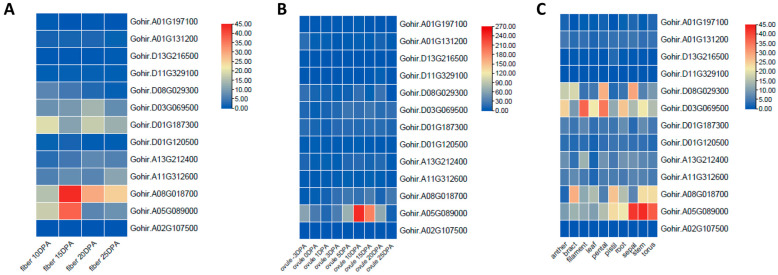
Expression profiles of *GhVLNs* in different tissues and organs in *Gossypium hirsutum* represented on a heatmap through TBtools. (**A**) fiber and (**B**) ovule at different (dpa), and (**C**) expression of *GHVLNs* in different tissues.

To investigate the potential functions of *GhVLNs* in various tissues of *Gossypium hirsutum*, we obtained tissue-specific expression data from the CottonRNA Database (PRJNA490626). The tissues examined included anthers, bracts, filaments, leaves, petals, pistils, roots, sepals, stems, ovules on different days post-anthesis (dpa), and fibers at different dpa stages (10, 15, 20, and 25 dpa). An analysis of the expression profiles of the 14 *GhVLN* genes revealed distinct spatial-expression patterns. For instance, Gohir.A05G089000 exhibited notably high expression levels in sepals, stems, and tori, whereas Gohir.D03G069500 showed elevated expression levels in filaments, petals, and roots (Figure 12C).

In ovule samples at different developmental stages, Gohir.A05G089000 showed the most prominent expression. In the case of fiber samples at various developmental stages, Gohir.A08G018700 had the highest expression at 10, 15, and 20 dpa, followed by Gohir.A05G089000, indicating its potential role in cell-wall thickening during fiber development. Gohir.A05G089000 was particularly expressed in 3-dpa ovules (Figure 12B).

**Figure 13:**
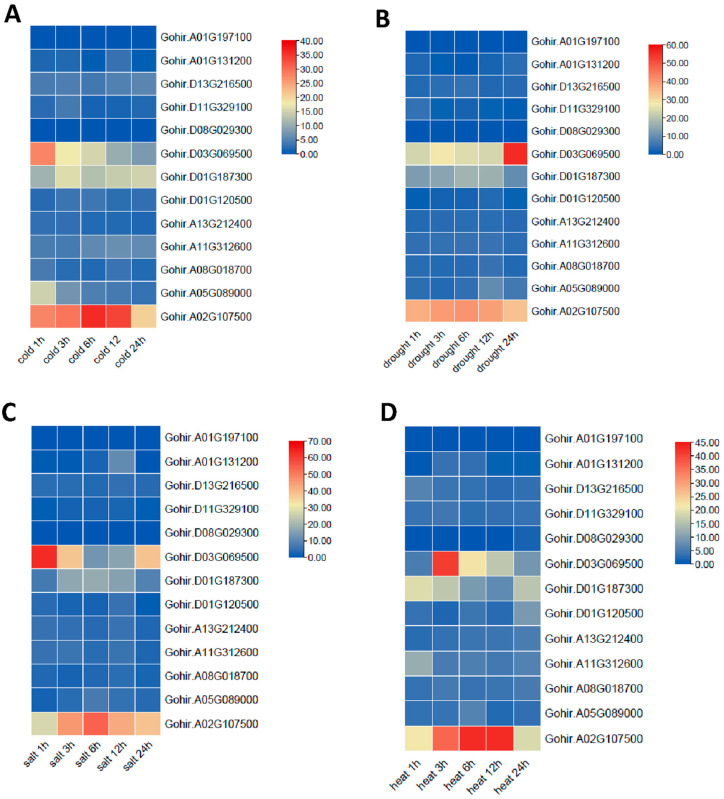
Expression patterns of *GhVLN* genes in response to different stresses analyzed from FPKM values at five time points (1, 3, 6, 12, and 24 h) after stress treatment. (**A**) Cold stress; (**B**) drought stress; (**C**) Salt stress; and (**D**) Heat stress.

Furthermore, Gohir.A02G107500, followed by Gohir.D03G069500, was found to be associated with stress responses, including cold, drought, heat, and salt environments (Figure 13). These genes could be considered candidates for future transformation experiments aimed at understanding their roles in cotton-fiber development.

## 4. Discussion

The *VLN* gene family plays a crucial role in various aspects of plant growth and development, and possesses an actin-binding domain. It acts as a group of regulators that control actin dynamics by polymerizing and depolymerizing actin filaments [54]. In different developmental stages and under the influence of environmental stress, VLN can modify actin filaments by either severing or bundling them [31,54]. Despite this, prior to our study, there has very limited investigation made for the *VLN* gene family in *Gossypium hirsutum*, a significant and widely cultivated fiber and cash crop. This genome-wide identification and characterization of the *Gossypium hirsutum VLN* gene family represents a vital initial step towards a deeper understanding of the functions of this gene family.

Several studies have investigated the functions of *VLN* genes in the regulation of plant architecture, which directly affects crop yields. These *VLN* genes control the development of various plant tissues by orchestrating the arrangement of actin filaments in *Arabidopsis thaliana* [5,31]. Notably, functional redundancies exist in *AtVLN2* and *AtVLN3*, both of which jointly influence the plant architecture. The simultaneous mutation of *VLN2* and *VLN3* results in distorted roots, stems, leaves, pods, and inflorescences [31,31]. In agricultural settings, twisted organs can adversely affect photosynthesis, biomass, and harvest. Moreover, *AtVLN5* plays a pivotal role in regulating pollen germination and pollen tube growth during the reproductive phase [31]. In light of these findings, it is evident that *VLNs* play a crucial role in the regulation of crop yield, offering insights into the genetic foundations of crop improvement.

In our investigation, we identified and characterized 14 Villin protein sequences in *Gossypium hirsutum* (Figure 1B). These genes have varying lengths, ranging from 902 to 980 amino acids, with molecular weights exceeding 100 kDa (Figure 1B). Subcellular localization analysis indicated their presence in the nucleus (Figure 2), which is consistent with the findings of previous studies on AtVLNs [55]. The amino acid alignment of the GhVLNs proteins represents the highly conserved nature of the protein sequences represented through distinct colors and through WebLogo (Figure 3 and Figure 4), respectively. The phylogenetic analysis of different species of *Gossypium* demonstrated that all VLNs could be grouped into three subcategories, A, B, and C (Figure 5), according to the clades dividing from the midroot point, which were unevenly spread across the tree. 

In our analysis of phylogenetic relationships across various model species and families, there were five distinct subcategories, A, B, C, and D, divided according to the midroot point (Figure 6). It was observed that the majority of GhVLNs were clustered within subgroups C and D (Figure 6). This trend was consistent not only within *Gossypium hirsutum* but also among other higher plants, including *Arabidopsis thaliana*, *Zea mays*, and *Oryza sativa*. Interestingly, VLNs from the lower plant *Chlamydomonas reinhardtii*, although fewer in number, were predominantly distributed in subgroup B. Remarkably, animalia species were consistently grouped in subgroup A (Figure 6). This pattern suggests a certain level of functional conservation in the gene family across higher plants, encompassing both monocots and dicots, as well as animalia species, which share some common ancestry with fungal and algal species. Our investigation further revealed that the majority of *Gossypium hirsutum* VLNs contained six gelsolin domains (G1–G6) and a villin-headpiece domain (VHP) (Figure 1A) which was verified by the conserved domains of the GhVLNs (Figure 7C). Additionally, 20 sequences were identified, the majority of which belong to the ADF and gelsolin superfamily (Table 1) (Figure 7B). These structural configurations align with the findings of previous studies [56,57].

The collinearity analysis using the *Arabidopsis* genome suggested that the synteny between the *GhVLN* gene and *Arabidopsis* may share a common ancestor during its evolution (Figure 8A). The 3D-structure prediction suggested that the GhVLNs exhibit similar spatial structures which were consisted with previous studies on *Glycine max* [57]. Furthermore, we employed protein–protein interaction and cis-element analysis to assess the gene expression pattern within the promoter region [58]. This analysis revealed the existence of diverse hormone- and stress-responsive elements in the promoter regions of *GhVLN* genes (Figure 11). The protein–protein interaction revealed that GhVLN proteins interact with various cytoskeletal proteins, hence playing a crucial role in stress responses (Figure 10). This observation implies that these genes likely play crucial roles in response to stress, aligning with similar findings in other species [56,57]. This outcome is in line with findings from other studies, where *AtVLNs* exhibited distinct expression patterns featuring elevated expression levels and a preference for specific tissues [59]. Furthermore, *GhVLNs* are broadly expressed in various tissues and organs. This widespread expression implies a significant role in regulating the stress tolerance, growth, and development of *Gossypium hirsutum*. Enhancing stress responses is a pivotal strategy for enhancing crop agronomic traits and increasing yield. This includes bolstering resistance to high temperatures, cold, salt, and drought, and responsiveness to hormones. Notably, Gohir.A02G107500, Gohir.D03G069500, Gohir.A05G089000, and Gohir.D03G069500 were primarily linked to stress responses and were expressed during different developmental stages (Figure 12 and Figure 13). Our analysis of cis-elements indicated that *GhVLNs* may participate in various processes, including hormone signaling and responses to light, drought, and defense mechanisms (Figure 11). Furthermore, a particularly intriguing observation captured our attention: specifically, the presence of pathogen-related cis-elements (W box) and wound-response elements (WUN-motif) within the promoter regions of certain *GhVLNs* (Figure 11), all of which were significantly induced under various biotic and abiotic stress treatments. Moreover, previous research has documented their involvement in the response to salt and drought stress, as well as their essential role in conferring tolerance to *Verticillium* infection in cotton. [60]. Notably, the high expression of Gohir.A025G089000 in sepals, stems, and torus suggests its potential involvement in the regulation of growth and elongation traits, which are crucial factors for *Gossypium hirsutum* yield regulation (Figure 12). The roles of the five *Villins* in *Arabidopsis* have been thoroughly characterized, and the *GhVLNs* investigated in this study are phylogenetically related to *Arabidopsis VLN4*, which is known to be involved in root-hair growth through the regulation of actin organization [5]. *GhVLN4* exhibits ubiquitous expression in cotton tissues, with its predominant presence in elongating young fibers. Previous reports have highlighted the crucial roles of *GhVLN4* in both tip and diffuse growth through the regulation of actin organization [27]. It has also been shown that VLN is directly involved in the developmental regulation of root hairs in plants under osmotic stress [30,61]. Our study revealed a high expression of Gohir.A08G018700 during fiber development from 15DPA to 25DPA (Figure 12A), indicating its involvement in the fiber-elongation phase, which is also consistent with some existing studies [56]. Given that cotton is primarily cultivated for its fiber yield, *Villin* genes play a crucial role in fiber elongation and development. To breed high-quality, high-yield *Gossypium hirsutum* varieties in the future, it is imperative to explore the function of the *VLN* gene family in shaping plant architecture and crop yield. Despite numerous studies on plant *VLNs* related to their role in regulating plant growth, development, and response to environmental stresses, our understanding of *Gossypium hirsutum VLNs* remains at an early stage.

Therefore, more investigations are required to assess and validate the effect of *VLN*s on the growth and development of *Gossypium hirsutum*. The present study of a genome-wide analysis may lay the groundwork for further exploration into the functions of *Gossypium hirsutum VLN*s, potentially offering valuable insights that could contribute to cotton breeding efforts.

## 5. Conclusions

The present study investigated the phylogeny and characteristics of the *VLN* genes in *Gossypium hirsutum* from various perspectives. All 14 GhVLN protein sequences were categorized into three distinct groups within the *Gossypium* species. Sequences within the same group exhibit comparable evolutionary features, such as three-dimensional structure, gene structure, motifs, and conserved domains, suggesting similar potential functions. Moreover, these *GhVLN* genes were distributed across 12 of the 26 chromosomes in the *Gossypium hirsutum* genome, and their amino acid sequences displayed a high degree of similarity. The collinearity analysis suggested that *GhVLNs* share functional similarities with *VLN* genes in the model species *Arabidopsis*. An examination of expression patterns across various tissues indicated that *GhVLN* genes are extensively expressed in different tissues, and that there may be functional overlaps among different *GhVLN* members. An analysis of protein-interaction networks and cis-elements suggests that *GhVLNs* may be involved in various physiological processes, including responses to hormones, stress, and developmental signals. The insights obtained from our study lay a robust foundation for future studies on the function of *VLN* genes in *Gossypium* and other higher plant species.

## Figures and Tables

**Table 1:** Characteristics of the 20 conserved motifs in GhVLNs.

Motif	Motif Sequences	Length (A.A)	Domain
1	VPFARSSLNHDDVFILDTQNKIYQFNGANSNIQERAKALEVVQFJKEKYH	50	ADF_gelsolin super family
2	YDIHFWJGKDTSQDEAGAAAIKTVELDAVLGGRAVQHRELQGHESDKFLS	50	ADF_gelsolin super family
3	FKVEEVYNFSQDDLLTEDILILDTHAEVFVWVGQCVDTKEK	41	ADF_gelsolin super family
4	SLEGLSPEVPJYKVTEGNEPCFFTTFFSWDSTKATVHGNSFQKKLALLFG	50	ADF_gelsolin super family
5	KGLLENNKCYLLDCGAEVFVWVGRNTQVEERKAASQAAEDF	41	ADF_gelsolin super family
6	LFRISGTSPHNMKAJQVDAVATSLNSSECFJLQSGSS	37	ADF_gelsolin super family
7	DYFLCCWIGKDSIEEDQKTAVRLANKMVN	29	Not identified
8	KGRPVQGRVFEGKEPPQFIAJFQPMVVLKGGLSAGYKKSIAEKGJTDETY	50	ADF_gelsolin super family
9	KVLDPAFQGAGQKPGTEIWRIENFQPVPLPKSDYGKFYMGDSYIVLQTTP	50	ADF_gelsolin super family
10	PPLLEGGGKMEVWCINGSAKTPLPKEDIGKFYSGDCYIVLYTYHSGERKE	50	ADF_gelsolin super family
11	YERLKASSTBPVTGIDVKRREAYLSDEEFKEKFGMEKEAFYKLPKWKQBK	50	Villin-headpiece domain
12	GTCEVAIVEDGKLDTESDSGEFWVLFGGFAPJPKKTASEDD	41	Not identified
13	WHGNQSTYEQQQLVARVAEFJKPGVQLKHAKEGSESNAFWSALGGKTEYT	50	ADF_gelsolin super family
14	RITRVIZGYETNSFKSKFDSWPQGSNAPGGEEGRGKVAALL	41	Not identified
15	YFKPCIIPLEGGVASGFKKPEEEEFETRLYVCRGKRVVKLK	41	Not identified
16	GPRQRAPALAALASAFNPSSASKTSAPKPVSRKQGSQRAAA	41	Not identified
17	EKESSEIVRDPHLFTFSFNKG	21	Not identified
18	TAEKKKQSPDGSPIKSTSSTPAVTSPPTEAKS	32	Not identified
19	KEEPQPYIDCTGNLQVWRVNGQEKVLLPA	29	ADF_gelsolin super family
20	TPAKLYSITDGEVKPVEGELS	21	Not identified

**Table 2:** Ka/Ks values for *GhVLN* collinear gene pairs.

Locus_1	Locus_2	Ka	Ks	Ka/Ks	Time (MYA)	Synteny	Selection
Gohir.D01G187300	Gohir.A01G197100	0.006674869	0.028485622	0.234324135	1.56514409	D01/A01	Purifying
Gohir.D03G069500	Gohir.A02G107500	0.008274678	0.054163656	0.152771775	2.976025047	D03/A02	Purifying
Gohir.D01G120500	Gohir.A01G131200	0.005862153	0.046331088	0.126527414	2.545664167	D01/A01	Purifying
Gohir.D11G329100	Gohir.A11G312600	0.013325515	0.043621315	0.305481738	2.396775561	D11/A11	Purifying
Gohir.A05G089000	Gohir.D05G090000	0.010134474	0.03314541	0.305757995	1.821176385	A05/D05	Purifying
Gohir.D08G029300	Gohir.A08G018700	0.004066581	0.042012538	0.096794467	2.308381209	D08/A08	Purifying
Gohir.A13G212400	Gohir.D13G216500	0.011020325	0.057520859	0.191588331	3.160486746	A13/D13	Purifying

## Data Availability

All supporting data are either provided with main text or as Supplementary Files.

## Supplementary Materials

The following supporting information can be downloaded at: https://www.mdpi.com/article/10.3390/cimb46030146/s1, Table S1: Protein sequences of all 94 VLNs in *Gossypium* species (*Gossypium hirsutum*, *Gossypium arboreum*, *Gossypium barbadense*, *Gossypium darwinii*, *Gossypium herbaceum*, *Gossypium mustelinum*, *Gossypium rainmondii*, and *Gossypium tomentosum*); Table S2: Protein sequences of all 115 VLNs, including *Gossypium hirsutum* and VLNs from other representative models from plants, algae, fungi, and animal species; Table S3: Detailed information regarding protein structure predictions, including metrics such as coverage, Global Model Quality Estimation (GMQE), and identity scores for the three-dimensional prediction of GhVLNs proteins using SWISS-MODEL; Table S4: Detailed information regarding protein–protein interactions, including predicted locus, function, and score metrics.
